# Celecoxib Inhibits Vasculogenic Mimicry and Induces Apoptosis in the D17 Canine Osteosarcoma Cell Line via the COX-2/PGE2 Signaling Axis

**DOI:** 10.3390/vetsci13030288

**Published:** 2026-03-19

**Authors:** Jungwoo Jo, Jungyun Kim, Jin-Young Chung, Jung-Hoon Choi, Yunho Jeong, Jin-Ok Ahn

**Affiliations:** 1 Department of Veterinary Internal Medicine, Institute of Veterinary Science, College of Veterinary Medicine, Kangwon National University, Chuncheon 24341, Republic of Korea; j3184432@naver.com (J.J.); 8house@naver.com (J.K.); jychung77@gmail.com (J.-Y.C.); 2 Department of Veterinary Anatomy, Institute of Veterinary Science, College of Veterinary Medicine, Kangwon National University, Chuncheon 24341, Republic of Korea; jhchoi@kangwon.ac.kr

**Keywords:** celecoxib, canine osteosarcoma, D17 cells, vasculogenic mimicry, prostaglandin E2, cyclooxygenase 2

## Abstract

Osteosarcoma is a highly aggressive canine bone tumor characterized by early metastasis and resistance to chemotherapy. Vasculogenic mimicry (VM), the ability of tumor cells to form microvascular channels independent of endothelial cells, can contribute to tumor progression and poor prognosis. In this in vitro study, we evaluated the effects of celecoxib, a selective cyclooxygenase-2 (COX-2) inhibitor, on the canine osteosarcoma cell line D17. Celecoxib treatment significantly inhibited cell proliferation in a dose- and time-dependent manner, induced S-phase cell cycle arrest, and promoted apoptosis. Furthermore, celecoxib effectively disrupted VM formation on Matrigel. Transcriptome analysis revealed that celecoxib downregulated genes associated with angiogenesis and the COX pathway, notably *PTGS2*. Consistent with this, celecoxib treatment reduced the secretion of prostaglandin E2 (PGE2) in a dose-dependent manner. Crucially, the addition of exogenous PGE2 restored VM formation in celecoxib-treated cells, confirming that celecoxib-mediated VM suppression is dependent on the reduction of PGE2 levels. These findings establish the COX-2/PGE2 signaling axis as a key regulator of VM in D17 canine osteosarcoma cells and that celecoxib warrants further preclinical evaluation as a strategy to target both tumor growth and alternative vascularization.

## Simple Summary

Osteosarcoma is an aggressive bone cancer in dogs that often spreads and shortens life expectancy. Tumors can form their own blood-carrying channels, a process called vasculogenic mimicry, which helps them grow and resist treatment. In this laboratory study, we tested celecoxib, a commonly used anti-inflammatory drug, on canine osteosarcoma cells to see whether it could reduce cancer cell survival and block formation of these channels. We found that celecoxib decreased cancer cell growth, increased programmed cell death, and prevented the cells from making blood-like channels. It worked by blocking a specific protein (COX-2) and reducing the production of a molecule (PGE2) that helps the tumor grow. These results suggest celecoxib may have potential as an additional treatment to slow tumor growth and reduce spread in dogs, but further studies in animals are needed to confirm safety and effectiveness before clinical use.

## 1. Introduction

Canine osteosarcoma (OSA) constitutes the predominant form of primary bone neoplasia in dogs, comprising nearly 85% of malignant skeletal tumors and about 5–6% of all canine cancers [1,2]. It most often affects middle-aged to older dogs (mean age: 7 years) and is particularly common in large and giant breeds such as Rottweilers, Great Danes, Saint Bernards, Irish Setters, Doberman Pinschers, German Shepherds, and Golden Retrievers. Although local control of OSA can be achieved surgically (for example, by amputation), microscopic metastases are frequently present at diagnosis, necessitating systemic chemotherapy. Despite this, meaningful improvements in disease-free interval (DFI) and overall survival remain limited, and metastasis continues to be a pressing clinical challenge [1].

Tumor growth and metastasis rely heavily on an adequate supply of oxygen and nutrients. Historically, angiogenesis—the formation of new blood vessels from pre-existing vasculature—was considered the primary mechanism for tumor blood supply. Consequently, anti-angiogenic therapies have been extensively investigated; however, their clinical efficacy has often been unsatisfactory [3,4]. These limitations suggest alternative blood-supply mechanisms, such as vasculogenic mimicry (VM) [5]. VM describes the ability of aggressive tumor cells to form fluid-conducting microvascular channels lined by tumor cells rather than endothelial cells [5,6,7]. VM channels have been identified in several aggressive human cancers, including osteosarcoma, glioblastoma, and breast cancer, and are associated with high-grade malignancy, metastasis, and poor prognosis [5,8,9,10,11]. In veterinary medicine, VM has also been observed in canine inflammatory mammary cancer and in canine osteosarcoma cell lines, such as D17 [12,13]. Because VM-rich regions are often resistant to conventional anti-angiogenic agents, targeting VM represents a promising therapeutic strategy [6,14].

Cyclooxygenase-2 (COX-2) has emerged as an important regulator of tumorigenesis and VM formation [15,16,17]. COX-2, the inducible isoform of cyclooxygenase, metabolizes arachidonic acid to produce prostanoids, including prostaglandin E2 (PGE2), a potent mediator of inflammation and tumor promotion [18,19]. COX-2 overexpression is common in many human and canine cancers, including in 77% of canine osteosarcomas, and is associated with increased proliferation, resistance to apoptosis, enhanced angiogenesis, and shorter survival times [20]. Previous studies have demonstrated that COX-2 regulates VM formation in human breast cancer cells, where high COX-2 expression correlates with extensive VM structures [15]. Furthermore, PGE2, the principal downstream product of COX-2, promotes tumor invasion and angiogenesis, implicating it in VM regulation [15].

Given the aggressive behavior of canine OSA and the limitations of current therapies, elucidating the mechanisms that drive VM formation is critical. Although COX-2’s role in tumorigenesis is well established, its specific contribution to VM formation in canine osteosarcoma cells remains unclear. In this study, we evaluated the antitumor effects of celecoxib, a selective COX-2 inhibitor, on the D17 canine osteosarcoma cell line. Specifically, we investigated whether celecoxib inhibits VM formation and whether such inhibition is mediated through the suppression of the COX-2/PGE2 signaling pathway.

## 2. Materials and Methods

### 2.1. Cell Cultures and Reagents

D17 cells, a canine osteosarcoma cell line, were purchased from the American Type Culture Collection (ATCC; Manassas, VA, USA). Cells were maintained in high-glucose Dulbecco’s Modified Eagle’s Medium (H-DMEM; Gibco, Aidenbach, Germany) supplemented with 10% fetal bovine serum (FBS; Gibco) and 1% penicillin–streptomycin (PS; 100 U/mL penicillin and 100 µg/mL streptomycin; Gibco) at 37 °C in a humidified atmosphere with 5% CO_2_. Celecoxib (Sigma-Aldrich, St. Louis, MO, USA) was dissolved in dimethyl sulfoxide (DMSO; Sigma-Aldrich) at a concentration of 100 mM and stored at −20 °C. For all experiments, the final DMSO concentration in culture medium was ≤0.1%.

### 2.2. Cell Viability Assay

The antiproliferative effect of celecoxib was determined by a WST-1 assay (Biovision, Milpitas, CA, USA). D17 cells were seeded in 96-well plates (SPL Life Sciences, Pocheon, Republic of Korea) at a density of 1 × 10^3^ cells/well. After 24 h, cells were treated with increasing concentrations of celecoxib (20–100 μM) or vehicle control (DMSO). After 24, 48, and 72 h of incubation, 10 µL WST-1 reagent (10% of the total working volume) was added to each well, and plates were incubated for 2 h. The absorbance was recorded at 450 nm, with a reference wavelength of 650 nm, using a 680 Microplate Reader (Bio-Rad, Hercules, CA, USA). Half-maximal inhibitory concentration (IC_50_) values were calculated with GraphPad Prism version 11 software (GraphPad Software, San Diego, CA, USA).

### 2.3. Cell Cycle Analysis

D17 cells were seeded at a density of 4 × 10^5^ cells per well into 60 mm culture dishes and incubated overnight at 37 °C in a humidified atmosphere containing 5% CO_2_. The following day, culture media were replaced with fresh media containing celecoxib at concentrations of 0, 60, or 80 μM. Cells were then incubated for 24 or 48 h under the same condition. Following incubation, floating cells were collected from the supernatant into 15 mL conical tubes. Adherent cells were gently rinsed with 1 mL PBS (Gibco), aspirated to dryness, and detached using trypsin. After 4 min of incubation at 37 °C and 5% CO_2_, the trypsinized cells were combined with the floating cell fraction. Cell suspensions were centrifuged at 1200 rpm for 5 min at room temperature. The supernatant was discarded, and the cell pellet was resuspended in 1 mL PBS for washing, followed by a second centrifugation under the same conditions. Pellets were then resuspended in 0.3 mL PBS and transferred to 1.5 mL Eppendorf tubes. Next, 0.7 mL of ice-cold 100% ethanol was added slowly with gentle pipetting to achieve fixation. Fixed samples were centrifuged at 200× *g* for 5 min at 4 °C, and the ethanol was carefully aspirated. Cells were treated with 500 μL RNase A (400 μg/mL; Sigma-Aldrich; final concentration 200 μg/mL) and incubated for 1 h at room temperature. Subsequently, 500 μL propidium iodide (PI; 1 mg/mL; Sigma-Aldrich) was added, and samples were incubated for an additional 30 min at 25 °C in the dark. Flow cytometric analysis was performed using a FACS Aria II (BD Biosciences, Franklin Lakes, NJ, USA), and data were analyzed with FlowJo software version 7.6.5 (Tree Star, Inc., Ashland, OR, USA).

### 2.4. Apoptosis Analysis

D17 cells followed the cell cycle analysis protocol through the initial PBS wash and centrifugation step (1200 rpm, 5 min). After discarding the supernatant by aspiration, cell pellets were resuspended in 1 × binding buffer for a second wash, followed by centrifugation under identical conditions. Cells were then resuspended in 500 μL 1 × binding buffer. Fluorochrome-conjugated Annexin V (5 μL; BioVision, Mountain View, CA, USA) was added, and samples were incubated for 10 min at room temperature in the dark. Following incubation, cells were washed with 2 mL 1 × binding buffer, pelleted by centrifugation (1200 rpm, 5 min), and resuspended in 200 μL 1 × binding buffer. Propidium iodide (5 μL of 1 mg/mL stock) was added immediately prior to analysis. Flow cytometric acquisition was performed using a FACS Aria II, and data were analyzed with FlowJo software version 7.6.5 to quantify apoptotic populations.

### 2.5. Vasculogenic Mimicry Formation Assay

Cells were treated with celecoxib (0, 60, and 80 µM). After 24 h of treatment, cells were plated at a density of 2 × 10^4^ cells/well in 96-well plates precoated with 50 µL/well growth factor-reduced Matrigel (BD Biosciences), which was allowed to solidify at 37 °C for 45 min. After 9 h of incubation, VM tube networks were imaged using an inverted phase-contrast microscope. VM assays were conducted with three biological replicates (*n* = 3). Five random fields per well were imaged (×40 magnification, consistent exposure) and quantified in a blinded manner by two independent investigators. VM parameters were quantified with the ImageJ Angiogenesis Analyzer plugin (version 1.52v; National Institutes of Health, Bethesda, MD, USA), measuring: (i) master junctions (branching points where ≥ 3 tube segments intersect), (ii) master segments (tube lengths between two junctions), and (iii) meshes (closed loops formed by tube networks) [21]. For the cell viability assay on Matrigel, 10 µL WST-1 solution (10% of the total working volume) was added and cells were incubated for 2 h. Absorbance was measured at 450 nm with a 650 nm reference using a spectrophotometer.

### 2.6. mRNA Sequencing

Transcriptomic profiling was performed to explore gene expression in D17 cells following 48 h exposure to either 80 μM celecoxib or vehicle control. Prior to cell seeding, each well of a 24-well plate was coated with 0.1 mL of Matrigel. Cell suspensions containing 1.4 × 10^5^ cells in 1 mL were then added to the Matrigel-coated wells and incubated at 37 °C for 9 h. Each experimental group consisted of three biological replicates (*n* = 3). Total RNA from these replicates was pooled prior to sequencing to generate a representative composite sample for each condition. A fold change > 2 or <−2 (log2-transformed signal > 4) was considered significant. Total RNA was extracted using TRIzol reagent (Invitrogen, Carlsbad, CA, USA). RNA integrity was evaluated using an Agilent 2100 Bioanalyzer with RNA 6000 Nano Chip (Agilent Technologies, Amstelveen, The Netherlands), while RNA concentration was determined with a ND-2000 spectrophotometer (Thermo Inc., Wilmington, DE, USA). Sequencing libraries were prepared from control and treated RNA samples (500 ng each) using the QuantSeq 3′ mRNA-Seq Library Prep Kit (Lexogen, Vienna, Austria) per the manufacturer’s protocol. Briefly, reverse transcription was initiated by annealing an Illumina-compatible oligo-dT primer to the poly-A tails of mRNA transcripts. After degradation of the RNA template, second-strand synthesis was carried out using a random primer harboring an Illumina-compatible linker sequence at the 5′ end. The resulting double-stranded cDNA libraries were purified with magnetic beads to eliminate reaction residues, followed by PCR amplification to attach complete adapter sequences required for cluster formation. The amplified libraries were again purified to remove PCR components. Sequencing was conducted as single-end 75 bp reads on an Illumina NextSeq 500 (Illumina Inc., San Diego, CA, USA).

### 2.7. Bioinformatics Data Analysis

Sequencing reads generated from QuantSeq 3′ mRNA-Seq were mapped using Bowtie2 [22], with reference indices constructed from the CanFam3.1 genome assembly. The resulting alignment files were used for transcript reconstruction and quantification of expression levels. Read count data from the pooled samples were normalized using the quantile normalization method, and gene expression levels were compared between celecoxib-treated and control groups. Functional annotation and gene classification were carried out using the DAVID Bioinformatics Resources (http://davidbioinformatics.nih.gov/ accessed on 27 January 2024) and literature searches (PubMed, http://pubmed.ncbi.nlm.nih.gov/ accessed on 27 January 2024).

### 2.8. Measurement of Prostaglandin E_2_ Production

D17 cells were plated at a density of 4 × 10^5^ cells/well in 60 mm tissue culture dishes (SPL Life Sciences) and then treated with celecoxib at concentrations of 0, 60, or 80 μM. Conditioned media were harvested 48 h post-treatment, centrifuged (1200 rpm, 5 min) to eliminate floating cells, and stored at −70 °C until analysis. PGE_2_ levels were quantified using the Parameter^TM^ PGE_2_ assay kit (R&D Systems, Minneapolis, MN, USA) according to the manufacturer’s protocol. Absorbance was measured at 450 nm with 540 nm as the reference wavelength using a 680 Microplate Reader. The PGE_2_ concentration of each sample was calculated based on the standard curve.

### 2.9. Rescue Assay with Exogenous PGE_2_

To investigate the involvement of PGE_2_ in VM formation, D17 cells were exposed to celecoxib at concentrations of 0 or 80 μM, with or without supplementation of exogenous PGE_2_ (100 ng/mL). Cells were seeded at a density of 2.5 × 10^5^ cells per well in 24-well plates precoated with Matrigel. The formation of VM structures was examined at 7 and 24 h after plating using an inverted microscope, and the luminal area was quantified using Image J software.

### 2.10. Statistical Analysis

All values are expressed as mean ± standard deviation (SD). Differences among groups were evaluated by one-way analysis of variance (ANOVA) followed by Dunnett’s post hoc test for multiple comparisons. Statistical analyses were performed using GraphPad Prism version 11 software. A *p*-value of < 0.05 was considered statistically significant.

### 2.11. Ethics Statement

All experiments were performed using the established D17 canine osteosarcoma cell line; no human participants or live animals were involved, and therefore formal institutional ethical approval for human or animal research was not required. All work complied with institutional biosafety and laboratory guidelines.

## 3. Results

### 3.1. Celecoxib Exerts Cytotoxic Effects on D17 Cells

**Figure 1:**
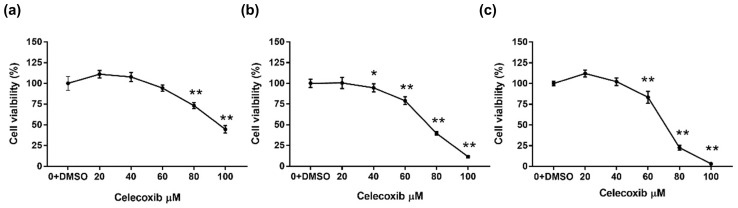
Inhibitory effect of celecoxib on D17 cell viability. D17 cells were treated with various concentrations of celecoxib for (**a**) 24 h, (**b**) 48 h, and (**c**) 72 h. Cell viability was determined by WST-1 assay and expressed as a percentage of control. Data are presented as mean ± standard deviation (SD). * *p* < 0.05, ** *p* < 0.0001 versus vehicle control by one-way ANOVA followed by Dunnett’s post hoc test.

To assess the effect of celecoxib on canine osteosarcoma cells, cell viability was evaluated using the WST-1 assay. The proliferation rate of the vehicle-treated control group was set as 100%. D17 cells treated with celecoxib showed a dose- and time-dependent decrease in viability compared to the control group (Figure 1). After 24 h of treatment, cell viability significantly decreased at concentrations of 80 and 100 μM (Figure 1a). After 48 h, cytotoxicity was significant at concentrations of 40–100 μM (Figure 1b). By 72 h, viability was significantly reduced at concentrations of 60–100 μM (Figure 1c). The IC_50_ values for 24, 48, and 72 h were 95.9, 74.14, and 70.64 μM, respectively, indicating a time-dependent increase in sensitivity to celecoxib.

### 3.2. Celecoxib Induces Cell Cycle Arrest in D17 Cells

**Figure 2:**
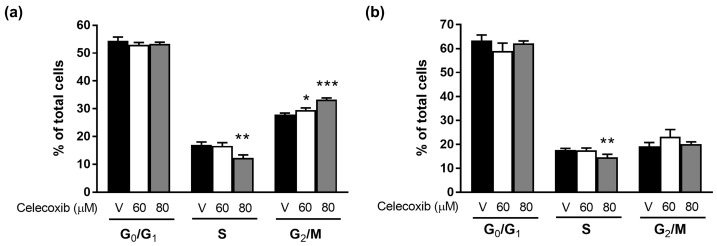
Effect of celecoxib on the cell cycle distribution of D17 cells. Cells were treated with various concentrations of celecoxib for (**a**) 24 h and (**b**) 48 h. The percentage of cells in each cell cycle phase was analyzed. Data are presented as mean ± standard deviation (SD). * *p* < 0.05, ** *p* < 0.01, *** *p* < 0.001 versus vehicle control by one-way ANOVA followed by Dunnett’s post hoc test.

To determine whether celecoxib’s antiproliferative effect resulted from cell cycle arrest, flow cytometry was performed on cells treated with 60 or 80 μM celecoxib for 24 and 48 h (Figure 2a,b). At 24 h, treatment with 80 μM celecoxib significantly decreased the S-phase population (from 17.05% to 12.31%, respectively, *p* < 0.01) and increased the G2/M-phase population compared to vehicle-treated controls. At 48 h, 80 μM celecoxib significantly reduced the S-phase population (17.59% to 14.57%, respectively, *p* < 0.01) with no significant change in G2/M phase. These findings indicate that celecoxib induces S-phase arrest in D17 canine osteosarcoma cells in a dose-dependent manner.

### 3.3. Celecoxib Induces Apoptosis in D17 Cells

**Figure 3:**
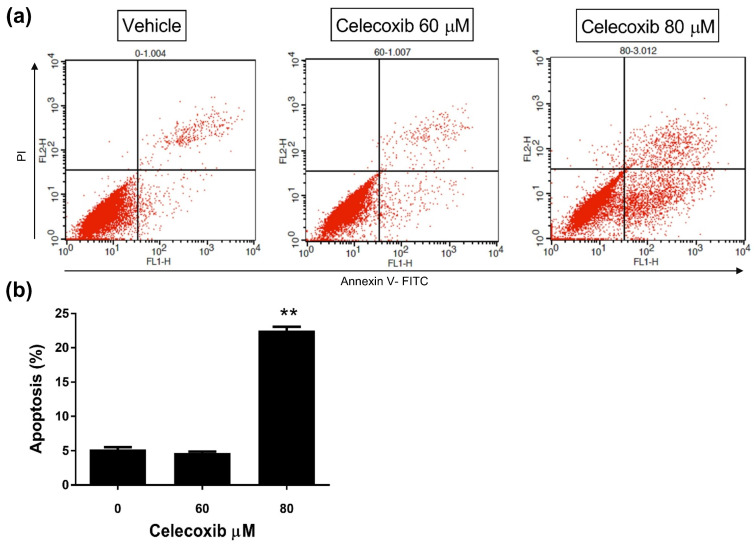
Induction of apoptosis by celecoxib in D17 cells. Cells were treated with celecoxib (0, 60, and 80 µM) for 48 h and stained with Annexin V-FITC/PI. (**a**) Representative flow cytometry plots showing apoptotic cell populations. (**b**) Quantitative analysis of the apoptotic rate. Data are presented as mean ± standard deviation (SD). ** *p* < 0.0001 versus vehicle control by one-way ANOVA followed by Dunnett’s post hoc test.

Annexin-V/PI staining was performed to evaluate apoptosis in D17 cells treated with celecoxib (60 and 80 μM) for 48 h (Figure 3a). The apoptotic population was defined as the sum of early apoptotic (Annexin V-positive/PI-negative) and late apoptotic (Annexin V-positive/PI-positive) cells (Figure 3b). The apoptosis rates for the control, 60 μM, and 80 μM groups were 5.18, 4.68, and 22.51%, respectively. Treatment with 80 μM celecoxib significantly increased the apoptotic rate by approximately 4-fold compared to the control (*p* < 0.0001). However, no significant difference was observed between the 60 μM group and the control.

### 3.4. Celecoxib Inhibits Vasculogenic Mimicry Formation in D17 Cells

**Figure 4:**
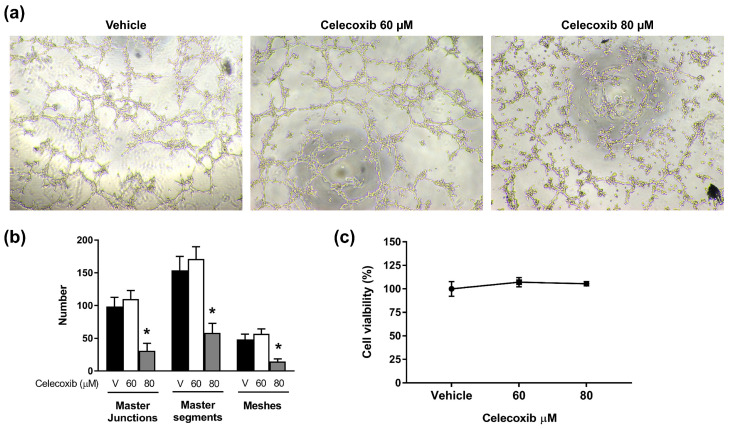
Effect of celecoxib on vasculogenic mimicry (VM) formation in canine osteosarcoma cells. (**a**) Representative images of tubule formation assays. Cells were treated with the indicated concentration of celecoxib. (**b**) Quantitative analysis of VM network parameters (master junctions, master segments, meshes) using ImageJ software. (**c**) Cell viability assay performed under the same experimental conditions to rule out cytotoxicity. Magnification, ×40. Data are presented as mean ± standard deviation (SD). * *p* < 0.0001 versus vehicle control by one-way ANOVA followed by Dunnett’s post hoc test.

Because COX-2 regulates various carcinogenic pathways, including angiogenesis, we investigated the effect of celecoxib on VM formation. D17 cells pretreated with celecoxib (0, 60, and 80 μM) were seeded on Matrigel and incubated for 9 h. Untreated D17 cells formed tube-like structures characteristic of VM; celecoxib inhibited these structures in a dose-dependent manner. At 60 μM, celecoxib did not significantly reduce the number of master junctions, master segments, or meshes compared to the control (Figure 4a,b). However, treatment with 80 μM celecoxib significantly reduced the number of master junctions, master segments, and meshes (*p* < 0.0001) (Figure 4a,b). To exclude cytotoxicity as the cause of VM disruption, cell viability on Matrigel was assessed. No significant difference in viability was observed between the control and celecoxib-treated groups at 9 h (Figure 4c), suggesting that celecoxib specifically inhibited VM formation rather than causing cell death.

### 3.5. Celecoxib Alters Expression of Genes Related to Angiogenesis and the COX Pathway

**Figure 5:**
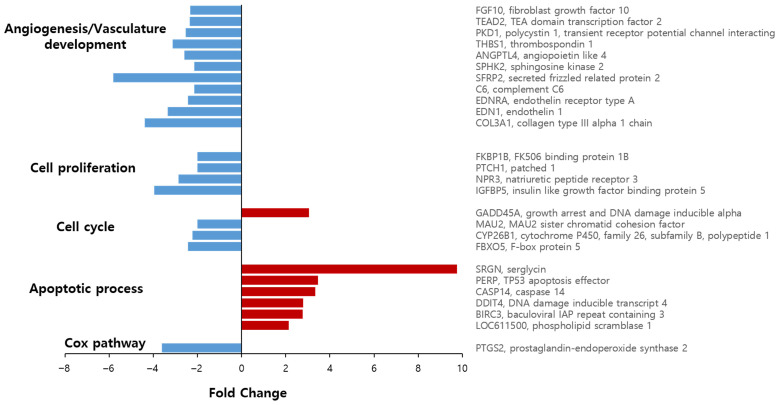
Differential gene expression analysis of celecoxib-treated D17 cells by mRNA sequencing. D17 cells were treated with or without 80 μM celecoxib for 48 h. Eleven angiogenic genes and one COX pathway gene were decreased after celecoxib treatment. Data represent fold changes relative to untreated controls.

mRNA sequencing was conducted to evaluate gene expression changes in D17 cells after celecoxib treatment. Of 31,296 genes analyzed, 145 were down-regulated and 205 were upregulated in the celecoxib-treated group base on fold-change criteria (>2-fold or <−2-fold with log2-transformed signal > 4). Genes involved in angiogenesis/vasculature development, cell proliferation, cell cycle, apoptosis, and the COX pathway were selected and plotted (Figure 5). Detailed fold-change values, normalized log2-transformed data, and raw read counts (RC) for these genes are provided in Supplementary Table S1.

Specifically, 11 genes related to angiogenesis and vasculature development were differentially expressed. Key angiogenic genes, including *FGF10* (fibroblast growth factor 10), *TEAD2* (TEA domain transcription factor 2), *ANGPTL4* (angiopoietin-like 4), *EDN1* (endothelin 1), *COL* (collagen), *PKD1* (polycystin 1), *THBS1* (thrombospondin 1), *SPHK2* (sphingosine kinase 2), *SFRP2* (secreted frizzled-related protein 2), *C6* (complement C6), and *EDNRA* (endothelin receptor type A), were down-regulated. Additionally, genes associated with cell proliferation (*FKBP1B*, *PTCH1*, *NPR3*, *IGFBP5*) were down-regulated, while the cell cycle-related gene *GADD45A* was up-regulated. Apoptosis-related genes (*SRGN*, *TP53*, *CASP14*, *DDIT4*, *BIRC3*, *PLSCR1*) were also up-regulated.

Notably, in the COX pathway, PTGS2 (prostaglandin-endoperoxide synthase 2), the gene encoding the enzyme responsible for PGE2 synthesis, was significantly down-regulated. Because PGE2 promotes carcinogenesis and VM, we focused on the suppression of PTGS2 as a potential mechanism for the observed VM inhibition.

### 3.6. Celecoxib Reduces PGE_2_ Production in D17 Cells

**Figure 6:**
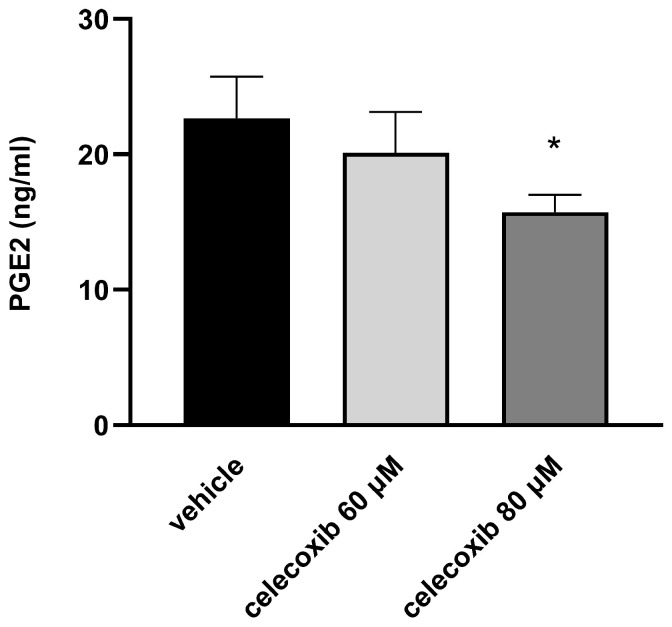
Celecoxib inhibits PGE_2_ production in D17 canine osteosarcoma cells in a dose-dependent manner. D17 cells were treated with celecoxib (0, 60, and 80 μM) for 48 h. PGE_2_ concentrations in the culture medium were quantified by ELISA. Data represent mean ± SD. * *p* < 0.01 versus vehicle control by one-way ANOVA followed by Dunnett’s post hoc test.

Because *PTGS2* expression was reduced by celecoxib, we measured PGE_2_ levels in the culture medium to confirm the functional impact. D17 cells were treated with celecoxib (0, 60, and 80 μM) for 48 h, and PGE_2_ concentrations were quantified by ELISA. As shown in Figure 6, the average PGE_2_ levels were 22.65 ± 3.08 ng/mL in the control group, 20.11 ± 3.02 ng/mL in the 60 μM group, and 15.74 ± 1.29 ng/mL in the 80 μM group. Celecoxib treatment resulted in a dose-dependent reduction in PGE_2_ production, consistent with the down-regulation of *PTGS2* observed in the mRNA sequencing data.

### 3.7. Exogenous PGE_2_ Restores Celecoxib-Inhibited VM Formation

**Figure 7:**
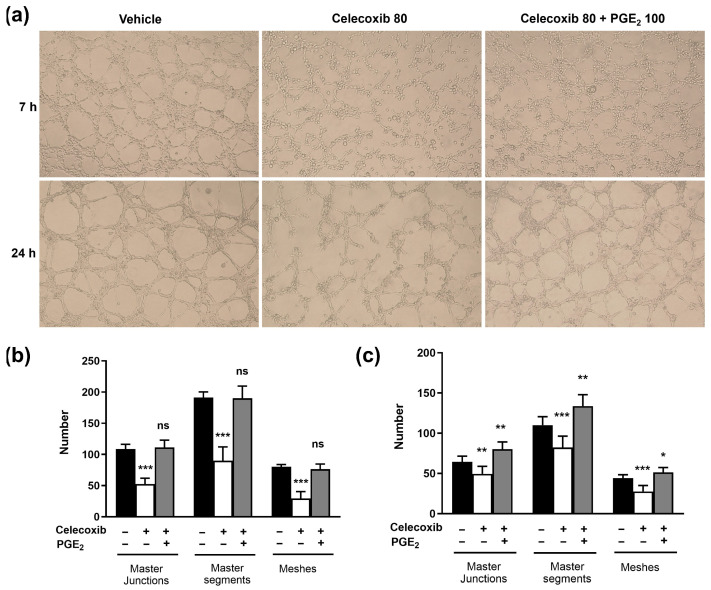
Exogenous PGE_2_ rescues celecoxib-inhibited vasculogenic mimicry formation in D17 canine osteosarcoma cells. (**a**) Representative phase-contrast images of VM formation in D17 cells treated with vehicle control, 80 μM celecoxib, or 80 μM celecoxib + 100 ng/mL PGE2 at 7 h and 24 h post-seeding on Matrigel. (**b**) Quantitative analysis of VM network parameters (master junctions, master segments, meshes) at 7 h using ImageJ software. (**c**) Quantitative analysis of VM network parameters at 24 h. Magnification, ×40. Data represent mean ± SD. * *p* < 0.05, ** *p* < 0.01, *** *p* < 0.001 versus vehicle control by one-way ANOVA followed by Dunnett’s post hoc test.

To confirm the role of PGE_2_ in VM formation, a rescue experiment was conducted by adding exogenous PGE_2_ (100 ng/mL) to cells treated with 80 µM celecoxib. As expected, 80 µM celecoxib alone significantly decreased the number of master junctions, master segments, and meshes compared to the control (Figure 7). However, the addition of 100 ng/mL PGE_2_ significantly restored VM formation in the presence of 80 µM celecoxib, increasing the number of master junctions, master segments, and meshes compared to the group treated with celecoxib alone. Notably, at 24 h, the PGE_2_-rescued group exhibited greater VM formation than the vehicle control, suggesting a stimulatory effect of exogenous PGE_2_. These results indicate that celecoxib-mediated suppression of VM formation depends on reduced PGE_2_ levels.

## 4. Discussion

In this study, we investigated the antitumor effects of the selective COX-2 inhibitor celecoxib on the D17 canine osteosarcoma cell line. Our results demonstrate that celecoxib exerts direct cytotoxic effects by inducing S-phase cell cycle arrest and apoptosis and that it significantly inhibits VM formation via suppression of the COX-2/PGE2 signaling pathway.

First, we confirmed the cytotoxic potential of celecoxib on D17 cells. Celecoxib produced a dose- and time-dependent reduction in cell viability, with an IC_50_ ranging from 70 to 96 µM. These findings are consistent with reports in human breast, cervical, and colon cancers, where celecoxib inhibited tumor cell proliferation [23,24]. Cell cycle analysis revealed that celecoxib induced S-phase arrest in D17 cells, which contrasts with reports of G0/G1 arrest in nasopharyngeal and bladder cancers [25,26] or G2/M arrest in HeLa cells [27]. This discrepancy suggests that the cell cycle modulatory effect of celecoxib may be cell-type-specific. In addition to cell-type-specific regulation, the observed difference may also reflect species-dependent variations between human and canine tumor cells. Celecoxib’s effects on cell cycle progression appear to be context-dependent, influenced by tumor origin, genetic background, and species. Canine osteosarcoma cells may differ from human carcinoma or cervical cancer cell lines in the expression patterns of COX-2, cyclins, CDKs, and p53, as well as in the downstream prostaglandin signaling pathways, which could shift the predominant arrest point from G_0_/G_1_ or G_2_/M to S-phase [28,29]. Furthermore, species-specific regulation of the COX-2/PGE_2_ axis and associated signaling networks might contribute to the distinct response observed in D17 cells [30,31]. While a detailed mechanistic comparison of human versus canine cell cycle regulation is beyond the scope of this study, this aspect warrants further investigation in future comparative studies.

Furthermore, we observed a significant increase in apoptosis at 80 µM, which was supported by the upregulation of apoptosis-related genes such as *TP53*, *CASP14*, and *SRGN* in our mRNA sequencing data. Together, these findings indicate that celecoxib-induced cytotoxicity in D17 canine osteosarcoma cells involves both cell cycle dysregulation and the induction of apoptotic pathways.

A key finding of this study is that celecoxib inhibits VM formation. VM is associated with poor prognosis and metastasis in various aggressive tumors [32,33,34]. We observed that D17 cells formed distinct VM tubular structures on Matrigel, as previously reported [13], and that 80 µM celecoxib significantly disrupted these VM structures. Importantly, cell viability on Matrigel at the 9 h time point used for the VM assay was not significantly affected by celecoxib, indicating that VM disruption was not a secondary consequence of acute cytotoxicity but rather a direct effect on the molecular processes that drive VM. While our morphological quantification (master junctions, segments, meshes) robustly demonstrated VM inhibition, molecular validation using VM markers (e.g., VE-cadherin, PAS staining) would further strengthen these findings.

To elucidate the underlying mechanism, we performed mRNA sequencing. Celecoxib treatment significantly downregulated genes associated with angiogenesis and vasculature development, including *COL*, *FGF10*, *TEAD2*, *ANGPTL4*, and *EDN1*. Downregulation of COL genes likely impairs VM by altering collagen-dependent three-dimensional extracellular matrix (ECM) architecture that supports tubular morphogenesis in collagen- and Matrigel-based VM assays [35,36]. Consistently, FGF10 and the broader FGF axis have been shown to stimulate endothelial tube formation on Matrigel and to prime vascular sprouting, while FGFR signaling is required for glioma VM-like structures in 3D culture [37,38]. TEAD2 functions as a transcriptional partner of YAP/TAZ; loss of YAP/TAZ–TEAD activity in endothelial cells leads to defective angiogenic growth, and YAP/TAZ have been implicated as central regulators of angiogenesis and tumor vascular mimicry [39,40]. Moreover, ANGPTL4 promotes vascular leakiness and pathological neovascularization by disrupting endothelial junctions and sustaining endothelial metabolism, whereas its inhibition stabilizes vessels and is associated with reduced angiogenesis and epithelial-to-mesenchymal transition signatures [41,42]. EDN1 (endothelin-1) is a potent pro-angiogenic and pro-invasive factor that drives endothelial sprouting and tube formation in glioma models; pharmacologic or genetic blockade of EDN1 attenuates tumor vascularization. Taken together, repression of these ECM-, growth factor-, and junction-related genes by celecoxib is mechanistically consistent with inhibition of VM, in addition to its well-described anti-angiogenic effects [43,44]. Most notably, *PTGS2* (encoding COX-2) was significantly downregulated. COX-2 promotes tumorigenesis and VM through PGE_2_ production in human cancer cells [15,17,45]. Consistent with the transcriptomic data, we found that celecoxib dose-dependently reduced PGE_2_ secretion in D17 cells. This reduction is critical, as PGE_2_ is a major mediator of inflammation, angiogenesis, and tumor progression [46,47].

We established a causal link between PGE_2_ reduction and VM inhibition using a rescue experiment. Addition of exogenous PGE_2_ completely restored VM formation in celecoxib-treated cells, providing strong evidence that celecoxib-mediated VM suppression depends on decreased PGE_2_. This finding is consistent with studies in human breast cancer and glioblastoma, where the COX-2/PGE_2_ pathway was shown to regulate VM via PKC/ERK signaling [15,17]. Our results suggest that COX-2-derived PGE_2_ is a crucial driver of VM in D17 canine osteosarcoma cells, and that its inhibition by celecoxib may represent a viable therapeutic strategy.

This study has certain limitations. First, experiments were performed in vitro, and the complex tumor microenvironment in vivo may modulate celecoxib’s efficacy; therefore, in vivo studies are needed to validate the relationship between COX-2 inhibition, PGE_2_ reduction, and metastasis suppression. Second, the study used only the D17 canine osteosarcoma cell line, and reliance on a single in vitro cell-line model limits generalizability because cell-line-specific genetics, phenotype, and culture adaptation can influence responses to celecoxib and VM capacity. While these findings establish a COX-2/PGE2-VM axis in D17 cells, validation in additional canine OSA lines (e.g., Abrams, HMPOS) and primary tumor cells is essential to determine the generalizability of this pathway across heterogeneous canine OSA. Third, RNA-seq analysis utilized pooled samples (*n* = 3 per condition), precluding replicate-based statistical testing and qPCR validation of candidate genes. Although fold-change screening identified key transcripts, independent qPCR confirmation across biological replicates would strengthen these transcriptomic findings. Fourth, although we focused on PGE2, other COX-2-derived prostanoids such as PGD2 or thromboxane A2 (TXA2) may also influence VM regulation and warrant further investigation. Finally, although we demonstrated PGE_2_ dependency, the precise downstream signaling events (for example, specific EP receptor subtypes and the PKC/ERK pathway) that link PGE_2_ to VM formation in canine cells remain to be fully elucidated.

Although pharmacologic inhibition with celecoxib reduced PGE2 secretion and VM formation, our evidence remains correlative and pharmacologic rather than definitively causal. Protein-level validation of COX-2 inhibition, enzymatic activity assays, genetic perturbation (e.g., CRISPR-Cas9 COX-2 knockout), or testing with alternative COX-2 inhibitors would be required to exclude off-target effects and establish pathway specificity—a priority for our ongoing follow-up studies.

## 5. Conclusions

In conclusion, our data demonstrate that celecoxib inhibited cell proliferation, induced S-phase cell cycle arrest, and promoted apoptosis in canine osteosarcoma D17 cells, thereby exerting potent antitumor effects. Celecoxib also disrupted VM formation in this cell line, a process driven by the COX-2/PGE_2_ signaling axis. COX-2 inhibition led to the downregulation of *PTGS2* expression and a subsequent reduction in PGE_2_ production, which directly mediated VM suppression. These findings establish COX-2 as a critical regulator of both tumor growth and vascular channel formation in this canine osteosarcoma model, suggesting that celecoxib could serve as a valuable therapeutic agent for targeting both primary tumor progression and metastasis associated with alternative vascularization. Further validation of our findings is warranted with in vivo and safety/pharmacokinetic studies before considering clinical translation.

## Data Availability

The RNA sequencing data supporting the findings of this study have been deposited in the NCBI Sequence Read Archive (SRA), accessible through BioProject ID: PRJNA1427609 (uploaded 24 February 2026) [https://www.ncbi.nlm.nih.gov/sra/PRJNA1427609 accessed on 28 February 2026].

## Supplementary Materials

The following supporting information can be downloaded at: https://www.mdpi.com/article/10.3390/vetsci13030288/s1. Table S1: Differentially expressed genes in celecoxib-treated D17 cells: fold-changes, normalized log2 signal values, and raw read counts
